# Vitamin D Status as a Risk Factor for Tuberculosis Infection

**DOI:** 10.1016/j.advnut.2025.100394

**Published:** 2025-02-20

**Authors:** Norah Tabsh, John P. Bilezikian

**Affiliations:** 1 Institute of Human Nutrition, Columbia University Medical Center, New York, NY, United States; 2 Division of Endocrinology, Department of Medicine, Vagelos College of Physicians and Surgeons, Columbia University, New York, NY, United States; 3 Mailman School of Public Health, Columbia University Medical Center, New York, NY, United States

**Keywords:** vitamin D, tuberculosis, infectious disease, nutrition, sunlight

## Abstract

Vitamin D is a principal regulator of bone and mineral metabolism. Recent data have provided evidence that vitamin D is a polyfunctional hormone with actions that extend beyond its classical role as a nutrient for bone and mineral metabolism. These additional actions include its potential role to prevent infections and autoimmunity. This review will focus on the relationship between vitamin D and infections, specifically on tuberculosis (TB). The literature review was conducted on PubMed using the search terms “Vitamin D” and “Tuberculosis.” As one of the most resilient infectious microorganisms on the planet, TB remains a public health concern because of the emergence of resistant strains, the burden, and side effects of treatment. Vitamin D plays an important role in the innate immune system that is the first line of defense against TB infection. Although there appears to be pathophysiological interplay between vitamin D and TB, more research is necessary to determine with certainty the extent of this relationship. Social determinants of health including population density, income, poverty, public assistance, unemployment, and education also play a role in estimating the prevalence of vitamin D deficiency and the burden of TB. This review explores the interplay between vitamin D and TB through factors including the immune system and social determinants of health.

**Statement of significance**

Putative, but controversial, nonskeletal effects of vitamin D include those related to immunity and infection. Conclusive evidence for or against these actions is lacking. One infection of particular historical and current interest is tuberculosis (TB). This narrative review is an up-to-date discussion on vitamin D and TB in which basic and clinical concepts are considered. With a historical background, this article explores vitamin D as a pleotropic nutrient and, in particular, as an antibacterial with particular emphasis upon TB. These observations underscore the potential public health significance of vitamin D as a risk factor for TB. Previous reviews exploring the relationship between vitamin D and TB have addressed vitamin D’s role in treatment and interventions. This review offers a novel insight by exploring potential associations in different populations, the public health significance of the deficiency and disease, and several other aspects of this relationship including immune function and socioeconomic status.

## Introduction

Vitamin D is a principal regulator of bone and mineral metabolism. When we refer to the actions of vitamin D, it is the active metabolite of vitamin D, namely 1,25-dihydroxyvitamin D (1,25OHD), not its relatively inactive precursors such as 25-hydroxyvitamin D (25OHD). The activation of vitamin D to 1,25OHD involves an intermediate metabolic step in the liver by which vitamin D (ergocalciferol—the plant form, or cholecalciferol, the animal form) is first converted to 25OHD in the liver. 25OHD is then hydroxylated in the kidney at the 1-position to form the active metabolite, 1,25OHD. When we refer to the actions of vitamin D, we are referring, unless otherwise indicated, to the active metabolite 1,25OHD. When we refer to circulating vitamin D levels, it is 25OHD that is the measured metabolite.

1,25OHD facilitates calcium and phosphate absorption in the gastrointestinal tract by 3 principal mechanisms: by stimulating the expression of TRPV6 calcium transporters on the apical membrane of the cell and the sodium-phosphate cotransporter type IIb; by increasing gene expression of calbindin to transport calcium within the cell; and by increasing the activity of calcium ATPase on the basolateral membrane and, thus, promoting paracellular diffusion of calcium through claudin 2 and claudin 12 proteins [1,2].

1,25OHD is a threshold nutrient regarding calcium absorption, which means that calcium absorption will increase as a function of increasing 25OHD concentration up to a threshold value beyond which further increases in 25OHD do not further increase calcium absorption. 1,25OHD also plays an important role in promoting skeletal mineralization both directly and indirectly, the latter by making substrates of bone mineral, namely calcium and phosphate, more available. A deficiency in 25OHD may lead directly to defects in bone formation (osteomalacia) and indirectly by a feedback loop leading to increased parathyroid hormone to increase bone resorption and bone loss. In children, defects in growth plate formation combined with 25OHD deficiency can lead to defects in bone formation, called rickets [1].

Recent data have provided evidence that vitamin D is a pleotropic hormone with actions that extend beyond its classical role as a nutrient for bone and mineral metabolism. These additional actions include its potential role to prevent infections and to prevent autoimmunity. In support of this point, vitamin D adequacy, as measured by 25OHD deficiency, is a risk factor when low for infections. Specifically, low 25OHD status has been implicated in risk of and severity of SARS-CoV-2 infection [3]. In addition, 25OHD status has been implicated as a risk of autoimmune and infectious diseases. This review will focus on the relation between vitamin D, as measured by 25OHD, and infections, specifically on tuberculosis (TB).

## Tuberculosis Significance and Background

**FIGURE 1:**
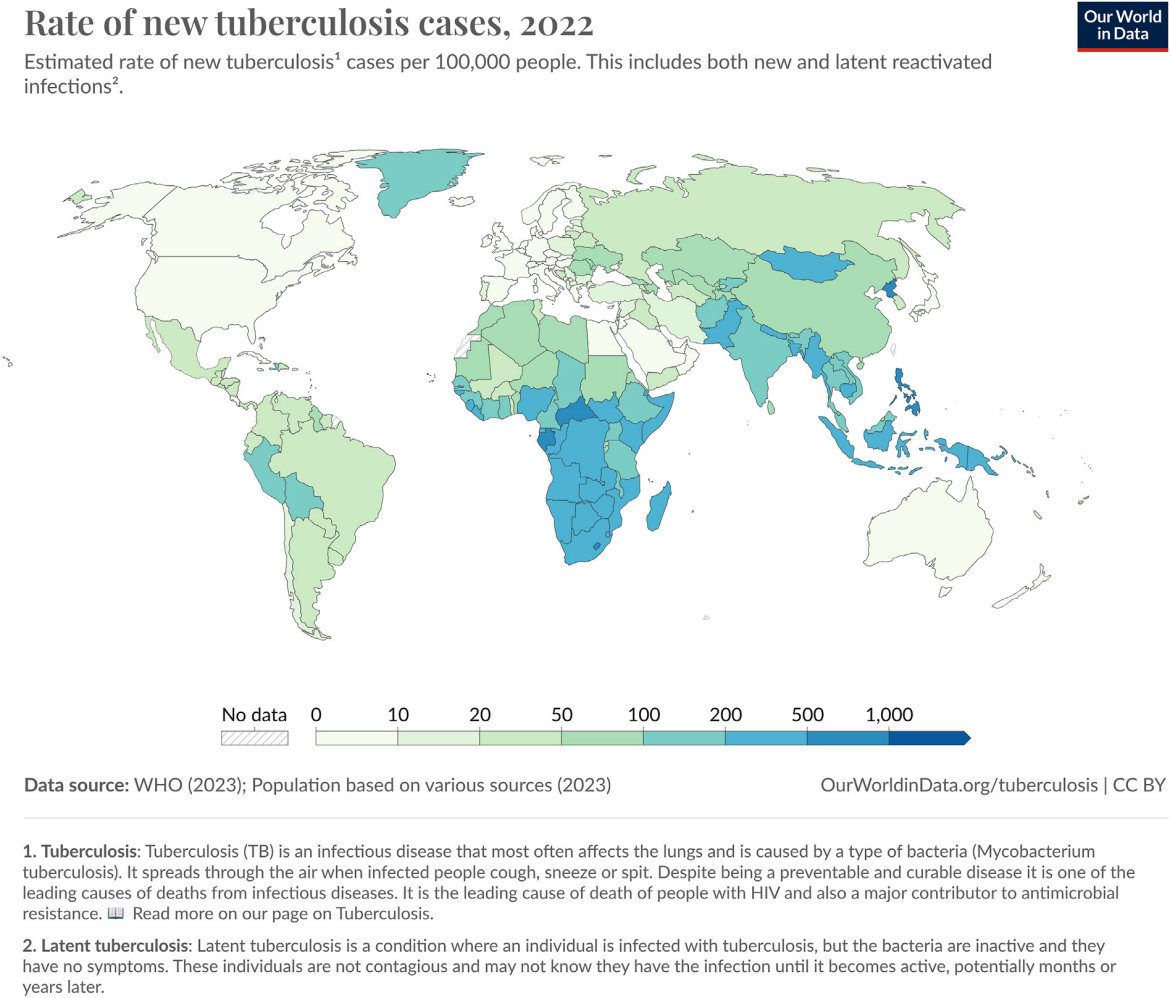
The global incidence rate of new and latent reactivated tuberculosis per 100,000 people in 2022 [6].

TB is an infectious disease caused by *Mycobacterium tuberculosis*. It is transmitted through aerosol droplets of an infected patient [4]. TB is one of the most resilient of infectious microorganisms on this planet. It has been around for ∼3 million years and can be traced back 9000 y in humans and even further back 17,000 y in animals [5]. The WHO declared TB a global health emergency in 1993, and despite its antiquity, TB continues to be a major public health concern. Figure 1 shows the incidence of new and latent cases of TB in 2022 [6]. In 2015, the WHO created a plan to eradicate TB with a goal to reduce by 75% the death and incidence rate by 2025, and by 95% by 2035 [7]. TB is particularly a concern in countries where sanitary conditions are substandard and where access to preventative healthcare is a challenge. In countries where TB is readily identified and treated, the organism has evolved with the emergence of resistant strains [4]. There are several factors that contribute to the emergence of resistant strains from a macro level including lack of government guidelines, poor supply of TB medications, and in fact the use of medications to treat the disease. On an individual level, lack of access and poor adherence to treatment, contribute, in part, to the emergence of resistant organisms [8]. Epidemiological factors are also important. The greater the number of people with resistant TB in a community, the greater the likelihood of resistant TB transmission. This is especially concerning with cases of undiagnosed, untreated, or incompletely treated TB [8]. Resistant TB, similar to wildtype strains, can remain latent for years and become active if and when the immune system is impaired. This poses a particular risk of those with impaired immune systems and with autoimmune diseases. Conditions such as diabetes can also contribute to risk. Lifestyle issue, such as smoking, is another risk factor [8]. These risk factors present a global challenge to limiting the impact of the disease, particularly in vulnerable communities where healthcare is substandard.

Before the discovery of antibiotics in the 1930s, 25OHD was implicated, in retrospect, as a therapy for patients with TB. In 1895, Niels Finsen was the first to use ultraviolet light to treat a form of skin TB called Lupus vulgaris, winning a Nobel Prize in 1903 [9]. In those early years, patients with TB were sent to sanatoriums, often located in the secluded countryside where sun was plentiful. Unwittingly, this approach, which was primarily designed to isolate individuals and to prevent spread, was also a therapy. Even before the advent of antibiotics to treat TB, there was a decline in studies investigating the use of 25OHD and other approaches to treat TB [10]. This may have been due, in part, to what appeared to be an effective therapeutic strategy to treat TB, namely exposure to fresh air (and sunlight).

1,25OHD has a major impact on all cells of the immune system. One aspect of 1,25OHD involvement in the immune system is through the activation of the innate immune system through the activation of IFN-γ and toll-like receptors in macrophages. IFN-γ and toll-like receptors allow the expression of Cyp27b1, which in turn allows macrophages to activate 1-alpha hydroxylase, the enzyme that catalyzes the conversion of 25OHD to the active metabolite, 1,25OHD [11]. Toll-like receptors also play a role in the expression of the vitamin D receptor (VDR), which is present in all cells of the adaptive and innate immune systems. VDR targets such as cathelicidin provide a first line of defense against foreign species by controlling the inflammatory response and activating the adaptive immune system [12,13]. The presence of variants of the gene Cyp27b1, which encodes for 1-alpha hydroxylase, the enzyme that catalyzes the conversion of 25OHD to the active metabolite, 1,25OHD, is associated with lower levels of 25OHD (<30 ng/mL) as well as with greater risk of TB. This was investigated in a study in South India where blood samples of 126 controls and 121 patients with asymptomatic pulmonary TB were collected, and polymerase chain reaction was used to genotype 3 polymorphisms of the Cyp27b1 gene [rs118204009 (G/A), rs118204011 (C/T), and rs118204012 (A/G)]. The study found that in rs118204012, the “A” allele and the “AA” genotype have higher susceptibility to pulmonary TB [odds ratio (OR): 1.52 (1.02, 2.26); *P* = 0.044; OR: 1.69 (1.02, 2.81); *P* = 0.040], and a higher prevalence of vitamin D insufficiency (66.67%) compared with controls (57.14%) [14]. The “G” allele in “AG” and “GG” genotypes of this gene variant were found to have protective factors against TB [OR: 0.66 (2.13, 9.79); *P* = 0.0001), thus suggesting that gene variants of Cyp27b1 may potentially be involved in downstream processing of serum 25OHD levels and TB. During TB infection, the bacteria encounter the innate immune system, the body’s first line of defense. However, as previously mentioned, the emergence of resistant strains has allowed TB to develop mechanisms to bypass the innate immune system [15].

## Vitamin D and Tuberculosis

Vitamin D3 is found in animal-based foods such as fish, cod liver oil, and egg yolk. Vitamin D2 is the predominant form of vitamin D in plants. Vitamin D3 is more effective than vitamin D2 in humans [16]. As either vitamin D3 or vitamin D2, it is also available in multivitamin supplements or as individual capsules of vitamin D3 or vitamin D2. As a fat soluble vitamin, vitamin D, from any source, is absorbed in the small intestine and then transported to the liver or through the lymphatic system into the circulation [17].

The evidence for the efficacy of vitamin D in preventing and/or treating TB is mixed. One of the earlier studies on the topic conducted by O’Riordan et al. investigated the efficacy of 1,25OHD, 1,24,25OHD, and 25OHD on survivability of monocytes in vitro that were infected with TB. Monocytes collected from blood samples were cultured with IFN-γ and these vitamin D metabolites and then infected with TB. Four days after TB exposure, these monocytes were lysed and the concentration of [3H]uracil incorporation into the TB bacteria was measured and presented as a dose-response curve. In this study, it was found that the presence of these vitamin D metabolites had an additive effect to that of IFN-γ. 1,25OHD inhibited [3H]uracil uptake when present in the concentration range of 10^−5^–10^−7^ M. 25OHD inhibited [3H]uracil uptake only in the highest concentration of 10^−5^M, and in the presence of 1,24,25OHD, there was intermediate inhibition of [3H]uracil uptake at 10^−7^M [18]. One study investigated the efficacy of vitamin D3 supplementation on 8851 children in a public school in Mongolia with a follow up 3 y later to measure TB infection. Children negative for TB were divided into 2 groups. An approximately equal number of children (4418 compared with 4433) were treated with 14,000 IU of vitamin D3 or placebo, respectively, weekly. Then they were tested again after 3 y. At the end of the study, it was found that 21 (0.5%) children in the vitamin D3 group tested positive for TB, and 25 (0.6%) children in the placebo group tested positive for TB. Compared with controls, the group that received vitamin D3 supplementation was not protected from TB infection [adjusted risk ratio, 1.10; 95% confidence interval (CI), 0.87, 1.38; *P* = 0.42] [19]. In contrast, a prospective case-control study of patients in India with both pulmonary and extrapulmonary TB found that the prevalence of TB in patients with vitamin D deficiency was 69%, whereas the group with vitamin D sufficiency showed a prevalence that was significantly lower at 52% (*P* = 0.029) [20]. Although the difference between these 2 groups was significant, the prevalence of TB among those who were vitamin D sufficient was still noteworthy.

In addition to obtaining vitamin D through food, vitamin D is synthesized by the skin through the conversion of 7-dehydrocholesterol by UV light (at wavelength 290–310 nm) into parent vitamin D (cholecalciferol) [21]. The subsequent steps summarized above in which vitamin D is activated by hydroxylation steps in the liver and the kidney permit active vitamin D to bind to VDRs. That process triggers a series of cellular events that in turn have major regulatory consequences in the nucleus of the target cells [22]. The angle at which the necessary wavelength of UV light reaches the skin determines the degree to which the mechanism is activated.

The incidence of TB differs across populations. The Coronary Artery Risk Development in Young Adults (CARDIA) study prospective longitudinal study in the United States found that TB was reported twice as frequently in Black participants in comparison with White participants. The extent of factors that may contribute to this observation are areas to be investigated; however, social determinants of health (SDOH) may play a role in this observation through environmental and economic factors [23]. A more recent study analyzing United States data from a 10-y period (2011–2021) similarly found a higher incidence of TB in the female and male Black population in comparison with the White population. Additionally, there is a higher incidence of TB in Asian, Hispanic, and “other” populations [24]. To the extent that socioeconomic and environmental factors are a risk factor for TB, public health solutions that are centered on these factors could become key preventative measures.

SDOH are nonmedical factors that play a large role in health outcomes. Thus, research into SDOH to determine causes of disease burden and establish solutions is a step toward a goal to increase health equity. A study based on United States census data from 1990 found that the relative risk of TB increased at a constant rate in relation to a decline in socioeconomic status (SES). Risk factors associated with this observation include population density, income, poverty, public assistance, unemployment, and education [25]. Several studies have found a relationship between SES and 25OHD status, including a study in Portuguese children that found that those in higher SES families had more play time outdoors (*P* < 0.001) and took more vitamin D supplements (*P* < 0.001) in comparison with children from lower SES [26]. Another study in Ireland found that the SES of children played a significant role in 25OHD status (low SES compared with high SES, odds ratio 2.18, CI: 1.34, 3.53, *P* = 0.002) [27]. The results of these studies illustrate the importance of an interplay between SES and 25OHD status as well as TB burden. This can be seen through the amount of free time people have to spend outdoors (rather than working), the safety of neighborhoods that determines how much time people are able to spend walking outside, and the cost of vitamin D supplements in determining 25OHD status and thus the extent of TB burden.

This discussion has mostly featured the efficacy of vitamin D as a treatment or preventative of active TB; however, there is limited research on the efficacy of vitamin D on latent TB. This raises the question of whether 25OHD status plays a more significant role in influencing the primary infection, making it more evident, compared with the reactivation of latent TB. To address this, a retrospective case notes review was completed on 67 patients diagnosed with active or latent TB in a pediatric clinic between June 2004 and 2006. The patient's age ranged from 0.1 to 17 y (median of 8.4 y), with 53% of the patients being male. The patient population was made up of 38% Black African and 55% South Asian. The serum 25OHD, and alkaline phosphatase of the patients at the time of diagnosis and before TB treatment between the latent and active TB groups were compared. It was found that 24 (37.5% of all) patients had a 25OHD concentration of <20 nmol/L, with 12 (46%) in the active TB cases and 12 (32%) in the latent TB cases. In total, 31 (48.5% of all) patients had an insufficient 25OHD concentration of <75 nmol/L, with 13 (50%) in the active TB cases and 18 (47%) in the latent TB group. Only 9 (14% of all) patients were vitamin D replete with just 1 (4%) of patients being in the active TB group and 8 (21% of) patients being in the latent TB group [28]. TB status had no significant effect on 25OHD levels; however, there was significant seasonal variation between the 25OHD of children in the active TB group, with a lower mean 25OHD of 21.5 nmol/L [IQR: 11–32 nmol/L] in the November–April cases compared with the May–October cases, with a higher mean 25OHD of 41.7 nmol/L (IQR: 18–54 nmol/L, *P* = 0.02). There was no significant difference in seasons for the latent TB cases (*P* = 0.1) and no correlation was found between alkaline phosphatase and 25OHD levels (*r* = 0.18). The presence and lack thereof seasonal variations in the active compared with latent TB groups may be explained by insufficient 25OHD reserves resulting from limited sunlight in the United Kingdom between November and April, with higher TB rates in the summer because of the lag between restoring 25OHD levels and the activation of latent TB [28,29].

Tuberculin skin test is a method of detecting active, previous, or latent TB infection through an injection under the skin of a purified protein derivative of tuberculin. A study in Spain investigated how vitamin D status may affect tuberculin skin test findings in patients with latent TB. The study had 2 phases with the first being a cross-sectional study where patients exposed to someone with TB undergo a tuberculin skin test and serum 25OHD levels are collected. The second phase being a case-control study where 2 mo after the tuberculin skin test, the patients with a negative result undergo another skin test. The results of phase 1 found that serum 25OHD was not associated with latent TB; however, despite not being significant, it was found to be a protector against TB (OR: 0.82; 95% CI: 0.36, 1.87). In phase 2, vitamin D supplements were not found to be associated with tuberculin skin test conversion; however, 11 patients with positive tuberculin skin test conversion were found to have low mean 25OHD levels (17.5 ± 5.6 ng/mL), whereas 69.5% of control patients (57 out of 82), who were exposed to someone with TB, however tested negative for TB, also had a low mean 25OHD of 25.9 ±13.7 ng/mL (*P* = 0.041). Despite this, there was a significant association between 25OHD levels and tuberculin skin test conversion, with 0–19 ng/mL (OR: 1.00; 95% CI: 0.12, 2.66), 20–29 ng/mL (OR: 0.60; 95% CI: 0.12, 2.66), and ≥30 ng/mL (OR: 0.13; 95% CI: 0.000, 0.91), which may suggest that sufficient 25OHD protects against tuberculin skin test conversion [30].

## Conclusions

In this article we have reviewed vitamin D’s role in TB through the involvement of the immune system, which has shown to have protective factors in vitro, as well as the presence of polymorphisms in vitamin D regulating genes, which may vary TB outcomes. Reports of the efficacy of vitamin D supplementation in preventing TB in children were found to have varying results. Higher SES was found to be a factor in 25OHD status as depicted by more outdoor play time, and access to vitamin D supplements in children. Results of these studies illustrate the importance of the interplay between SES and 25OHD status as well as TB burden. There is limited research on the efficacy of 25OHD status on latent TB. However, the presence and lack of seasonal variations in active compared with latent TB groups may be explained by insufficient 25OHD reserves resulting from limited sunlight in winter, with higher TB rates in the summer. Last, a significant association between 25OHD levels and tuberculin skin test conversion may suggest that sufficient 25OHD protects against tuberculin skin test conversion. TB has been, is, and will continue to remain a major public health crisis for the foreseeable future. The remarkable longevity of this organism is due to its socioeconomic demographics as well as to the emergence of resistant strains. Vitamin D, which plays an important role in the innate immune system, is a first line of defense. Although there appears to be pathophysiological interplay between vitamin D and TB, more research is necessary to determine the pathophysiological and clinical ramifications of this relationship. The interplay between social determinants and concomitant vitamin D deficiency is also a factor that influences the extent of the TB burden. Despite valiant efforts by many public health agencies throughout the world, TB has proved itself to be resilient and impossibly hard to eradicate.

## Author contributions

Both authors read and approved the final manuscript.

## Funding

The authors reported no funding received for this study.

## Conflict of interest

The authors report no conflicts of interest.
